# A Comparison of Recruitment Methods for a Remote, Nationwide Clinical Trial for COVID-19 Treatment

**DOI:** 10.1093/ofid/ofae224

**Published:** 2024-04-29

**Authors:** Katrina M Hartman, Barkha Patel, Via Rao, Aubrey A Hagen, Hanna G Saveraid, Regina Fricton, Samuel Lee, Andrew T Snyder, Matthew F Pullen, David R Boulware, David M Liebovitz, Hrishikesh K Belani, Jacinda M Niklas, Thomas A Murray, Ken Cohen, Jennifer L Thompson, Spencer M Erickson, Carolyn T Bramante

**Affiliations:** General Internal Medicine, University of Minnesota, Minneapolis, Minnesota, USA; General Internal Medicine, University of Minnesota, Minneapolis, Minnesota, USA; General Internal Medicine, University of Minnesota, Minneapolis, Minnesota, USA; General Internal Medicine, University of Minnesota, Minneapolis, Minnesota, USA; General Internal Medicine, University of Minnesota, Minneapolis, Minnesota, USA; General Internal Medicine, University of Minnesota, Minneapolis, Minnesota, USA; General Internal Medicine, University of Minnesota, Minneapolis, Minnesota, USA; General Internal Medicine, University of Minnesota, Minneapolis, Minnesota, USA; General Internal Medicine, University of Minnesota, Minneapolis, Minnesota, USA; General Internal Medicine, University of Minnesota, Minneapolis, Minnesota, USA; General Internal Medicine, University of Minnesota, Minneapolis, Minnesota, USA; General Internal Medicine, Northwestern University, Chicago, Illinois, USA; General Internal Medicine, University of Minnesota, Minneapolis, Minnesota, USA; General Internal Medicine, University of Minnesota, Minneapolis, Minnesota, USA; General Internal Medicine, University of Minnesota, Minneapolis, Minnesota, USA; M Health Fairview, Minneapolis, Minnesota, USA; General Internal Medicine, University of Minnesota, Minneapolis, Minnesota, USA; Division of Infectious Diseases and International Medicine, University of Minnesota, Minneapolis, Minnesota, USA; General Internal Medicine, University of Minnesota, Minneapolis, Minnesota, USA; Division of Infectious Diseases and International Medicine, University of Minnesota, Minneapolis, Minnesota, USA; General Internal Medicine, University of Minnesota, Minneapolis, Minnesota, USA; General Internal Medicine, Northwestern University, Chicago, Illinois, USA; General Internal Medicine, University of Minnesota, Minneapolis, Minnesota, USA; Department of Medicine, Olive View - University of California, Los Angeles, California, USA; General Internal Medicine, University of Minnesota, Minneapolis, Minnesota, USA; General Internal Medicine, University of Colorado, Denver, Colorado, USA; General Internal Medicine, University of Minnesota, Minneapolis, Minnesota, USA; Division of Biostatistics, School of Public Health, University of Minnesota, Minneapolis, Minnesota, USA; General Internal Medicine, University of Minnesota, Minneapolis, Minnesota, USA; UnitedHealth Group, Optum Labs, Minnetonka, Minnesota, USA; General Internal Medicine, University of Minnesota, Minneapolis, Minnesota, USA; Department of Obstetrics and Gynecology, Vanderbilt University Medical Center, Nashville, Tennessee, USA; General Internal Medicine, University of Minnesota, Minneapolis, Minnesota, USA; General Internal Medicine, University of Minnesota, Minneapolis, Minnesota, USA; General Internal Medicine, University of Minnesota, Minneapolis, Minnesota, USA; General Internal Medicine, University of Minnesota, Minneapolis, Minnesota, USA

**Keywords:** decentralized clinical trial, mutli-site trial, early Covid-19 treatment

## Abstract

This study describes decentralized recruitment and enrollment for a COVID-19 treatment trial, while comparing 5 primary recruitment methods: search engine ads, paid advertising within a national testing company, paid advertising within a regional testing company, electronic health record messages, and word of mouth. These are compared across patient demographics, efficiency, and cost.

**Clinical Trials Registration:**

NCT04510194.

Decentralized methods provide an opportunity to recruit diverse populations, reduce environmental impact of research-participant travel, and reduce transmission of infectious disease [1]. However, little has been published on decentralized recruitment or the enrollment processes.

The COVID-OUT trial was a phase 3, multisite, decentralized clinical trial of early outpatient treatment of COVID-19 that remotely enrolled and consented participants. The objective of this paper is to describe the 5 primary recruitment methods and compare efficiency between them to improve optimal approaches for future decentralized trials.

## METHODS

COVID-OUT was a phase 3, quadruple-blinded, placebo-controlled randomized clinical trial of outpatient treatment of COVID-19 (ClinicalTrials.gov: NCT04510194) [2]. The trial enrolled participants recruited throughout the United States. There was a central institutional review board (IRB, Advarra), and each participating site's IRB reviewed and approved their local version of the consent.

### Participant Recruitment

The trial used 5 recruitment methods: search engine ads, paid advertising through a national COVID-19 testing company, a regional COVID-19 testing company, electronic health record messages, and placing flyers.

During the first month of the trial (January 2021), recruitment occurred via secure electronic health record (EHR) patient portal messages to patients of participating health care systems who had pending SARS-CoV-2 tests. After the first month of the trial, portal messages to patients were sent only to those with positive tests. A reporting workbench was set up to automatically generate a list of patients who fit basic criteria: (1) positive SARS-CoV-2 test result in the past 24 hours and (2) age 30-85 years. Secure EHR patient portal messages with basic study information were sent to those with activated portal accounts who had not opted out of research.

Early in the pandemic, the participating health systems allowed patients to also be cold-called if they had a positive test in the system. A health-system-run recruitment office called patients with positive SARS-CoV-2 tests to inform them about the trial, and if patients were interested, they would be re-called by a research coordinator on the study team for enrollment.

The principal investigator met with a patient advisory panel, and based on their feedback, the decision was made to remove the intermediate step of calling potential participants and then sending interested participants to the study team. Instead, the study team formally trained health system staff on the protocol so they could complete consent with interested participants during the initial outreach conversation. This seamless enrollment was essential to not losing potential participants, and to enrolling participants earlier in their infection.

All advertising of the study included the study website, which was optimized by purchasing a domain that would be easy for patients to remember (“COVIDout.com”). The website included a secure REDcap survey where interested patients could submit their contact information. When a survey was filled out, a message went to all research staff. Two study numbers (Spanish and English) were listed on the website. If a participant called a study phone number directly, a phone tree service would waterfall the call to the official study phones of available research coordinators. The study team sent the website to community health and charitable organizations that were helping to boost awareness of COVID clinical trials.

In July 2021, more methods were added including search engine ads, outreach to local newspapers and radio stations, posting community flyers with a QR code that went to the study website, and testing companies advertising to patients with positive test results. The search engine ads were triggered when individuals searched key phrases (ie, “I just tested positive for COVID-19”) and the study website would be at the top of the results. The testing companies included the study website at the bottom of the email sent to patients with a positive test.

### Screening

Eligibility was confirmed by a research coordinator asking the criteria over the phone; or participants could fil out a self-screening questionnaire via secure REDcap link from the study website (Supplementary Figure 1).

The study team kept secure call-back databases that were color-coded so coordinators would easily know which potential participants needed to be contacted about enrollment. For patients who consented, baseline data were then collected in the study's REDCap database.

**Figure 1.**
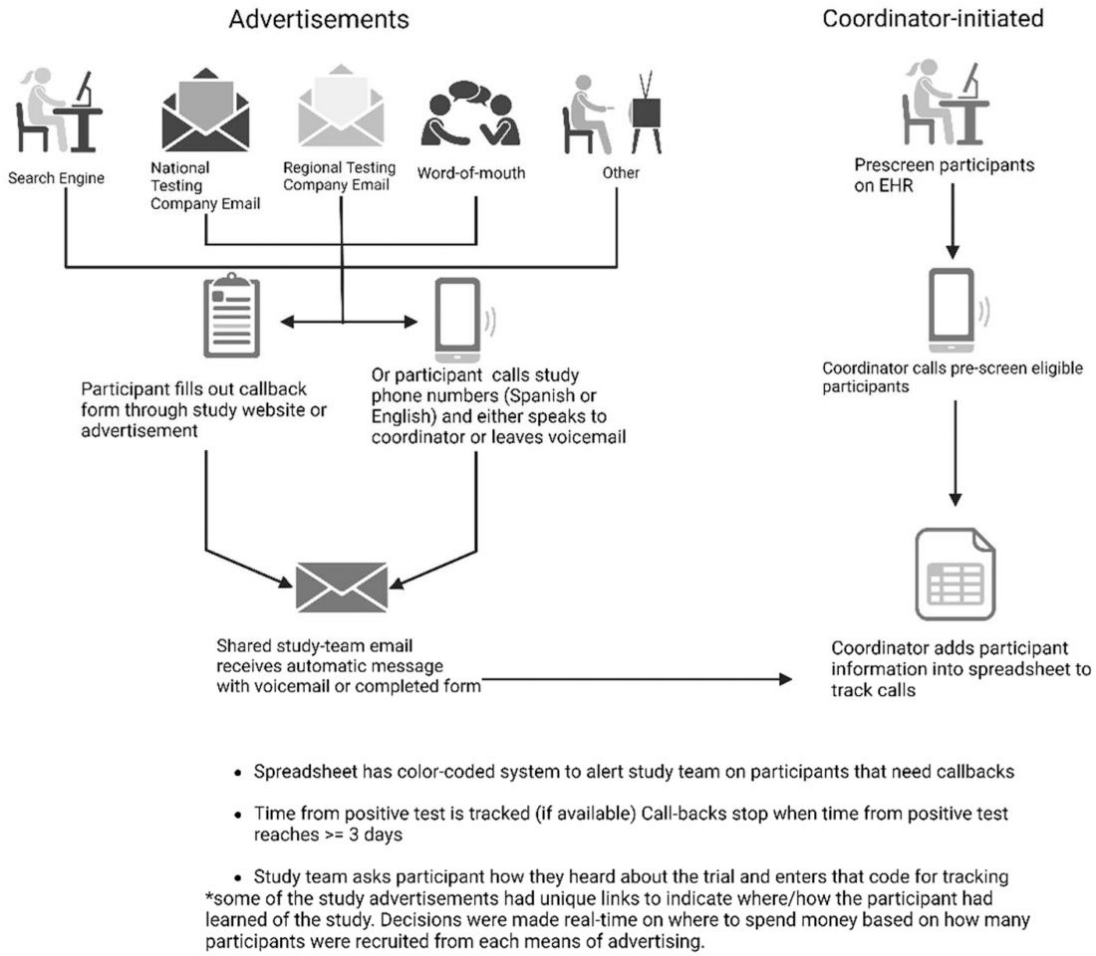
Recruitment strategies and overall research study enrollment process. (Created with BioRender.com.)

This process was later streamlined with an online REDcap self-screen form on the website that allowed potential participants to complete initial screening or complete eligibility screening and consent using a secure database link. Participants were also prompted with questions to assess their understanding and ability to provide informed consent. They could always reach out via phone call or email with questions before consenting. See Figure 1 for further details regarding recruitment process and flow.

### Remote Consent

Consent was obtained securely using a Part 11 remote consent platform that required a unique passcode for each participant.

### Statistical Methods

Descriptive statistics were used to compare participant characteristics between the recruitment methods. Efficiency is defined as the proportion enrolled out of the number screened by each recruitment method. All calculations were done in Excel.

## RESULTS

**Figure 2.**
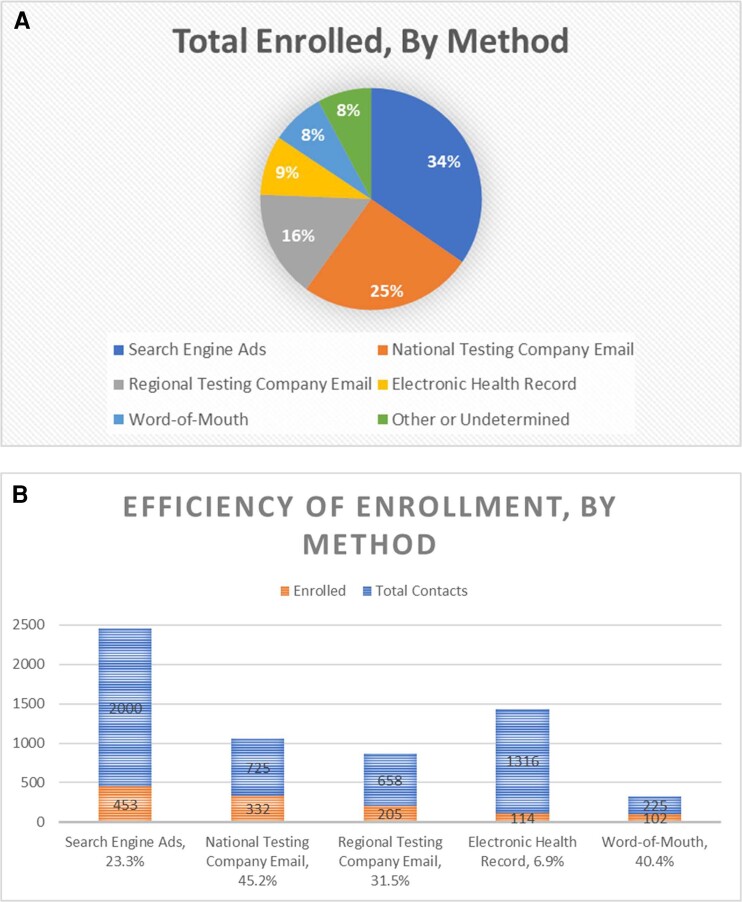
*A*, Distribution of participants enrolled by recruitment method. Total n = 1323. *A* demonstrates the distribution of participants enrolled by each recruitment method. *B*, Efficiency of enrollment by recruitment method. Total contacted n = 4995. Total enrolled shown n = 1206 because of n = 117 enrolled methods not obtained/recorded. *B* shows the efficiency of each recruitment method in gaining enrollments by percent efficiency and contact number. In these diagrams, only 1206 enrollees are depicted because of 117 enrollees’ methods of recruitment not being recorded.

Figure 1 outlines participant-initiated contacts (search engine, national testing company, regional testing company, word-of-mouth, other) and coordinator-initiated contacts (EHR). The “other” category included nonquantifiable (ie, flyers) or undetermined (ie, participant does not remember how they came across study) efforts. Overall, 453/1323 (34%) were enrolled from search engine ads, 332/1323 (25%) from a national testing company, 205 (16%) from a regional testing company, 114/1323 (9%) from the EHR, 102/1323 (8%) heard about the study through word of mouth, and a small percentage (8%) of enrollees were enrolled by other methods (ie, flyers, news articles in local print papers) or unknown (Table 1, Figure 2*A*).

**Table 1.** Baseline Characteristics of Enrollees, Overall and by Each Recruitment Method

Baseline Characteristics	Overall n = 1309	Search Engine Ads	National Testing Company	Regional Testing Company	Electronic Health Record	Word of Mouth	Other, or Undetermined
		n = 453	n = 332	n = 205	n = 114	n = 102	n = 117
Age, median (IQR)	45 (37, 54)	45 (37, 54)	45 (37, 55)	46 (37, 54)	46 (40, 58)	46 (38, 55)	45 (39, 53)
Female, n (%)	738 (56)	303 (67)	165 (50)	90 (44)	66 (58)	55 (54)	59 (57)
Race, n (%)							
Native American	30 (2.3)	10 (2.4)	6 (1.8)	5 (2.4)	2 (1.8)	2 (2.0)	5 (4.9)
Asian	49 (3.8)	13 (3.0)	18 (5.4)	10 (4.9)	1 (0.9)	3 (2.9)	4 (3.9)
Hawaiian, Pacific Islander	8 (0.6)	1 (0.2)	4 (1.2)	3 (1.5)	0 (0.0)	0 (0.0)	0 (0.0)
Black	95 (7.3)	39 (9.2)	29 (8.7)	8 (3.9)	5 (4.4)	5 (4.9)	9 (8.7)
White	1030 (79)	350 (82)	260 (78)	157 (77)	102 (89)	82 (80)	79 (77)
Other/declined/missing	97 (7.4)	40 (8.8)	15 (4.5)	22 (11)	4 (3.5)	3 (2.9)	4 (3.9)
Ethnicity, n (%) Latino	148 (11)	49 (12)	34 (10)	39 (19)	6 (5.3)	7 (6.9)	13 (13)
Medical history							
BMI, median (IQR)	30 (27, 34)	30 (27, 35)	30 (27, 33)	29 (27, 33)	31 (27, 35)	30 (27, 34)	30 (27, 34)
BMI > = 30 kg/m^2^	644 (49)	235 (52)	156 (47)	90 (44)	64 (56)	47 (46)	52 (50)
Cardiovascular disease*	451 (34)	136 (30)	115 (35)	67 (33)	71 (62)	29 (28)	33 (32)
Diabetes	28 (2.1)	14 (3.1)	3 (0.9)	4 (2.0)	6 (5.3)	0 (0.0)	1 (1.0)
Pregnant/breastfeeding	71 (5.4)	54 (12)	10 (3.0)	2 (1.0)	1 (0.9)	2 (2.0)	2 (2.0)
Vaccinated, primary series	666 (51)	210 (46)	210 (63)	124 (60)	29 (25)	50 (49)	43 (42)
Symptoms present on day 1	1288 (98)	450 (99)	321 (97)	204 (99)	109 (96)	101 (99)	103 (100)
Insurance							
Medicaid	199 (15)	107 (23)	33 (9.9)	18 (8.8)	11 (9.6)	17 (17)	13 (13)
Medicare	104 (7.9)	45 (9.9)	13 (3.9)	17 (8.3)	12 (11)	8 (7.8)	9 (8.7)
Private	789 (60)	225 (50)	231 (70)	128 (62)	84 (74)	60 (59)	61 (59)
No insurance	198 (15)	68 (15)	53 (16)	39 (19)	3 (2.6)	17 (17)	18 (17)

*Cardiovascular disease defined as: hypertension, hyperlipidemia, coronary artery disease, past myocardial infarction, congestive heart failure, pacemaker, arrhythmias, or pulmonary hypertension.

Search engine ads had the highest proportion of women enrolled (67%) compared to the national testing company (50%), the regional testing company (44%), EHR (58%), and word of mouth (54%). Search engine ads resulted in a higher proportion of black participants enrolled (9.2%) compared to advertisements in a regional testing company (3.9%) and EHR-based recruitment (4.4%). Regional testing company advertisements resulted in the highest proportion of Latinx participants enrolled (19%), compared to 5.3% from EHR messages (Table 1).

The median body mass index was similar across all methods. The EHR had the highest proportion of participants with body mass index ≥30 kg/m^2^ (56%). Of participants enrolled through the EHR, 62% had cardiovascular disease compared to 28% to 35% recruited from other methods (Table 1). Although most (60%) of participants had private insurance, regional testing company advertisements recruited 19% of participants with no insurance, compared to 2.6% from EHR recruitment.

Paid advertising in a national testing company appeared to be the most efficient, followed by word of mouth, paid advertising in a regional testing company, search engine ads, and EHR messages (Figure 2*B*).

**Figure 3.**
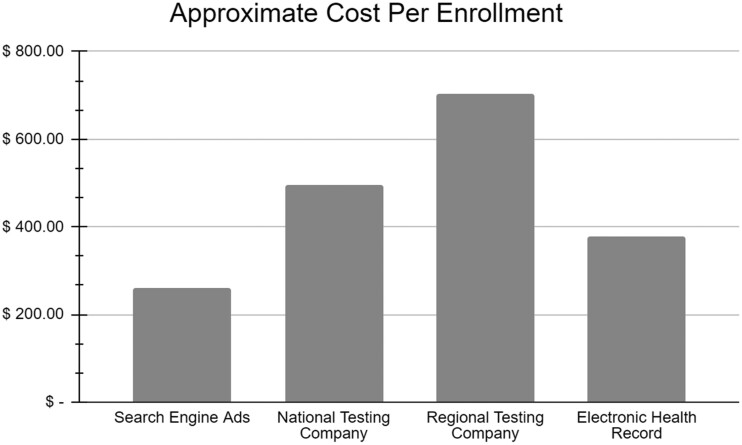
Cost per participant enrolled by recruitment methods. Of note, the electronic health record calculated costs are higher if counting health system research staff salaries once trained on protocol.

The regional testing company was the most expensive method, followed by national testing company, EHRs, and search engine ads. The costs are approximate, especially as the word of mouth and the other/undetermined category are not quantifiable (Figure 3).

## DISCUSSION

Clinical trials rely on efficient recruitment of representative participant samples. The methods used in this trial showed some differences in the populations recruited, but more data from several trials are necessary to understand whether some methods may be better. The proportion of participants enrolled in this trial from groups typically underrepresented in research (Hawaiian/Pacific Islander, Native American, Asian, Black, and Latinx) was low across all recruitment methods. Overall, there was underrepresentation of females, pregnant women, Black, and Asian participants in COVID-19 research, underscoring the need for tailored strategies to enhance inclusivity and representation in clinical trials [3].

However, it is important take measures to avoid manipulation of marginalized or vulnerable groups, such as only using IRB-approved material; having advertisements link directly to secure study databases; and to confirm all participants understand the risks and benefits of participation.

EHR-based recruitment was initiated by the study team, which may explain the lower proportion of participants enrolled from the EHR compared to outreach initiated by potential participants.

Although the national testing company was the most efficient recruitment method in enrollees per contact, it was also the second most expensive method in cost per enrollee, after the regional testing company. A clinical trial may have dynamic priorities with staffing, enrollment speed, specific patient population, and cost, and may need to adjust recruitment methods accordingly.

During the COVID-19 pandemic, several other trials used varied recruitment strategies to engage participants. Boulware et al. used primarily social media and traditional media methods, whereas Lenze et al. recruited from a larger variety, via EHR, health professional referrals, advertisements near testing centers and in emergency departments, a study website, and traditional media communications through television and newspapers [4, 5]. Other clinical trials describe recruitment as simply via self-identification or site identification [6].

This study has several limitations. Because some of the recruitment methods are specific to the COVID-19 pandemic (ie, companies focused on providing testing), these methods may not generalize beyond COVID-19.

In summary, various recruitment methods can be used, depending if the researcher wants to target efficiency, cost, or specific participant populations. Future research should describe the effectiveness of recruitment methods across different conditions and populations. In a global pandemic, multiple strategies should be used simultaneously.

## Supplementary Material

ofae224_Supplementary_Data

## Supplementary Data

Supplementary materials are available at *Open Forum Infectious Diseases* online. Consisting of data provided by the authors to benefit the reader, the posted materials are not copyedited and are the sole responsibility of the authors, so questions or comments should be addressed to the corresponding author.

## Note

***Potential conflicts of interest.*** All authors: No reported conflicts.
