# Supplementary material for: A Comparison of Recruitment Methods for a Remote, Nationwide Clinical Trial for COVID-19 Treatment
Source: Open Forum Infect Dis. 2024 Apr 29;11(7):ofae224. doi: 10.1093/ofid/ofae224 (PMC11214097; doi:10.1093/ofid/ofae224)
Supplement: ofae224_Supplementary_Data [file ofae224_supplementary_data.docx]

**Supplementary Figure 1.**

| **Initial eligibility criteria (Metformin only)**   - Ages 30-85 - Able to provide informed consent - English, Spanish, (or translated/approved material) speaking - Had a positive Covid test result within past 3 days - Never previously had Covid-19 diagnosis - BMI greater or equal to 25 kg/m^2 - Symptoms started within last 7 days (if any symptoms at all; symptoms not required) - Not currently taking metformin, insulin, cimetidine, hydroxychloroquine, sulfonylureas, dolutegravir, patiromer, ranolazine, or tafenoquine - No history of severe kidney disease, liver disease, or stage ¾ heart failure (GFR <45 mL/min in last 2 months or home phlebotomy arranged) - Not currently enrolled in another Covid treatment trial - No current alcohol use disorder (defined as >3 drinks/day average) - Not currently admitted to hospital, an inmate in penitentiary or inpatient rehabilitation center - No history of lactic acidosis - Not immunocompromised (bone marrow transplant, solid organ transplant, active cancer treatment, AIDs, high-dose steroids >20mg oral daily, etc) - No monoclonal antibody treatments already completed | **Criteria added later (after trial expanded to include ivermectin and fluvoxamine)**   - Ages 18-29 were added for metformin arm, if pregnant - No history of allergic reaction to metformin, ivermectin or fluvoxamine - No current Loa Loa or Onchocerciasis infection - No typhoid, BCG, or cholera vaccination within last 14 days or next 3 days - Not currently taking ivermectin or fluvoxamine - Not currently taking sodium picosulfate, rasagiline, selegiline, MAOIs, linezolid, duloxetine, methylene blue, tizanidine, ramelteon, alosetron, agomelatine, bromopride, dapoxetine, tasimelteon, thioridazine, urokinase, pimozide - No reported bipolar disorder or taking medication for bipolar disorder (lithium, valproate, high dose antipsychotic) - Not on a dose (or combination of doses) that is unsafe of the following medications (as evaluated by PI): SSRI, SNRI, tricyclic antidepressant, alprazolam, diazepam, theophylline, clozapine, olanzapine, NSAIDS, aspirin warfarin (also known as Coumadin), phenytoin, clopidogrel, St. John's wort |
| --- | --- |
